# Supplementary material for: A male-killing gene encoded by a symbiotic virus of Drosophila
Source: Nat Commun. 2023 Mar 13;14:1357. doi: 10.1038/s41467-023-37145-0 (PMC10011393; doi:10.1038/s41467-023-37145-0)
Supplement: Supplementary file 1 — Supplementary Information [file 41467_2023_37145_MOESM1_ESM.pdf]

Supplementary Information

A male-killing gene encoded by a symbiotic virus of *Drosophila*

Kageyama, Harumoto *et al.*

This pdf file contains:

Supplementary Figures 1–14

Supplementary Tables 1–2

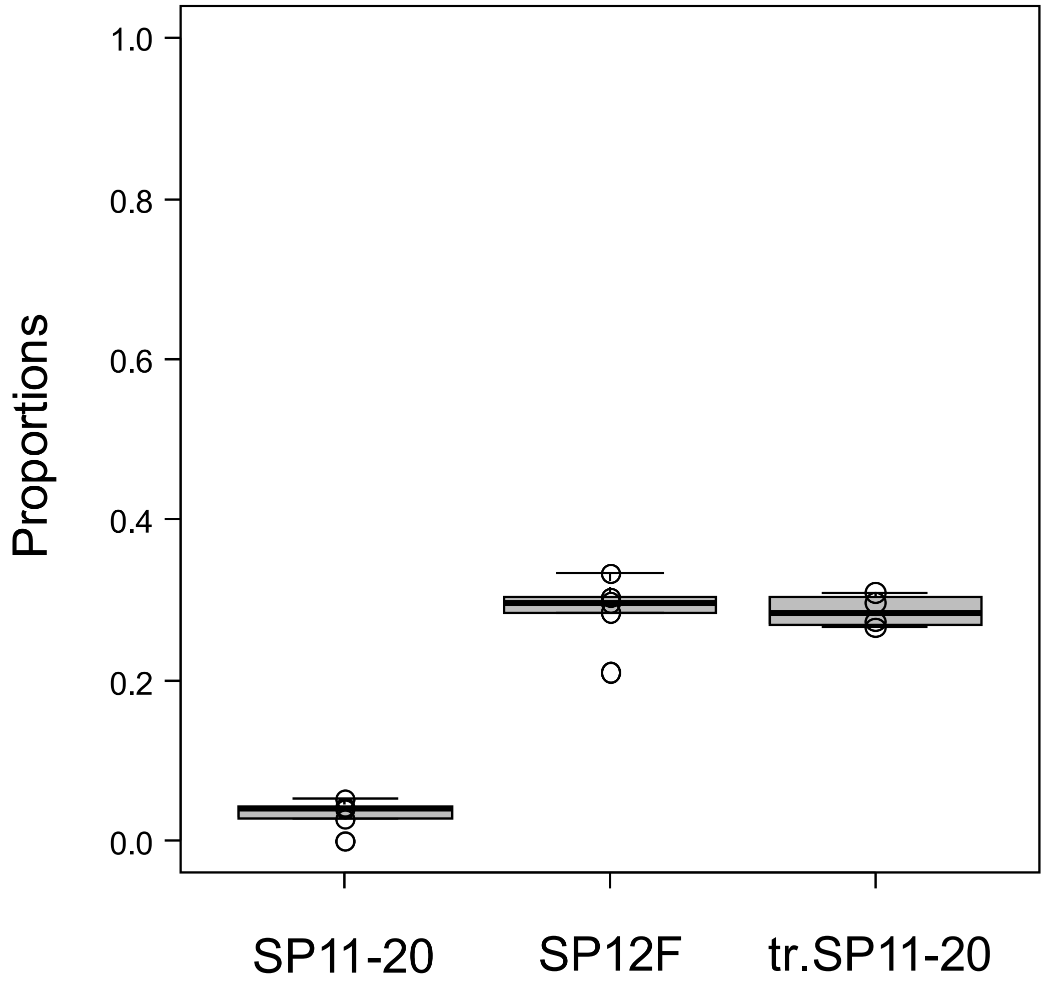

Supplementary Figure 1. Proportion of well-developed dead embryos per total number of eggs laid. Box plots represent the median, the first quartiles and third quartiles with whiskers drawn within the 1.5 IQR value. Points outside the whiskers are outliers. None or very few embryos died after development in SP11-20 (nonbiased line), but considerable number of such embryos were observed in SP12F and tr.SP11-20 (all-female-producing lines). SP11-20: n = 180, 152, 135, 373 and 879 eggs examined over 5 independent experiments. SP12F: n = 120, 135, 199, 217 and 810 eggs examined over 5 independent experiments. tr.SP11-20: n = 310, 754, 689 and 866 eggs examined over 4 independent experiments. Source data are provided in Supplementary Data 1.

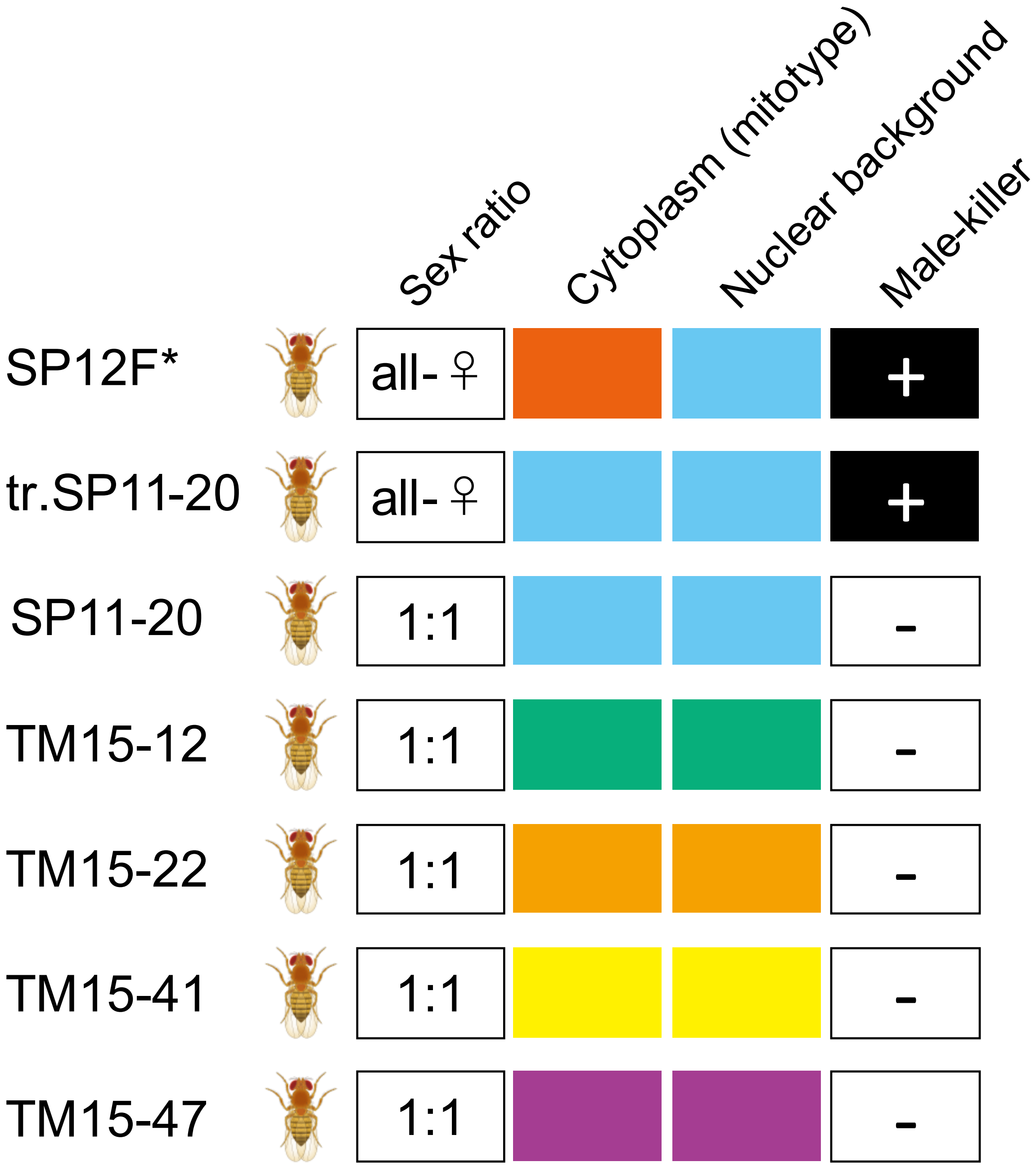

Supplementary Figure 2. Experimental design of RNA-seq for identification of the causal agent of all-female trait in *D. biauraria*. Seven matrilines differing in sex-ratio trait, cytoplasmic and nuclear genetic background were subjected to RNA-seq. *Because SP12F was backcrossed by SP11-20 males for more than 50 generations, nuclear background of SP12F, SP11-20, and tr.SP11-20 should be almost identical.

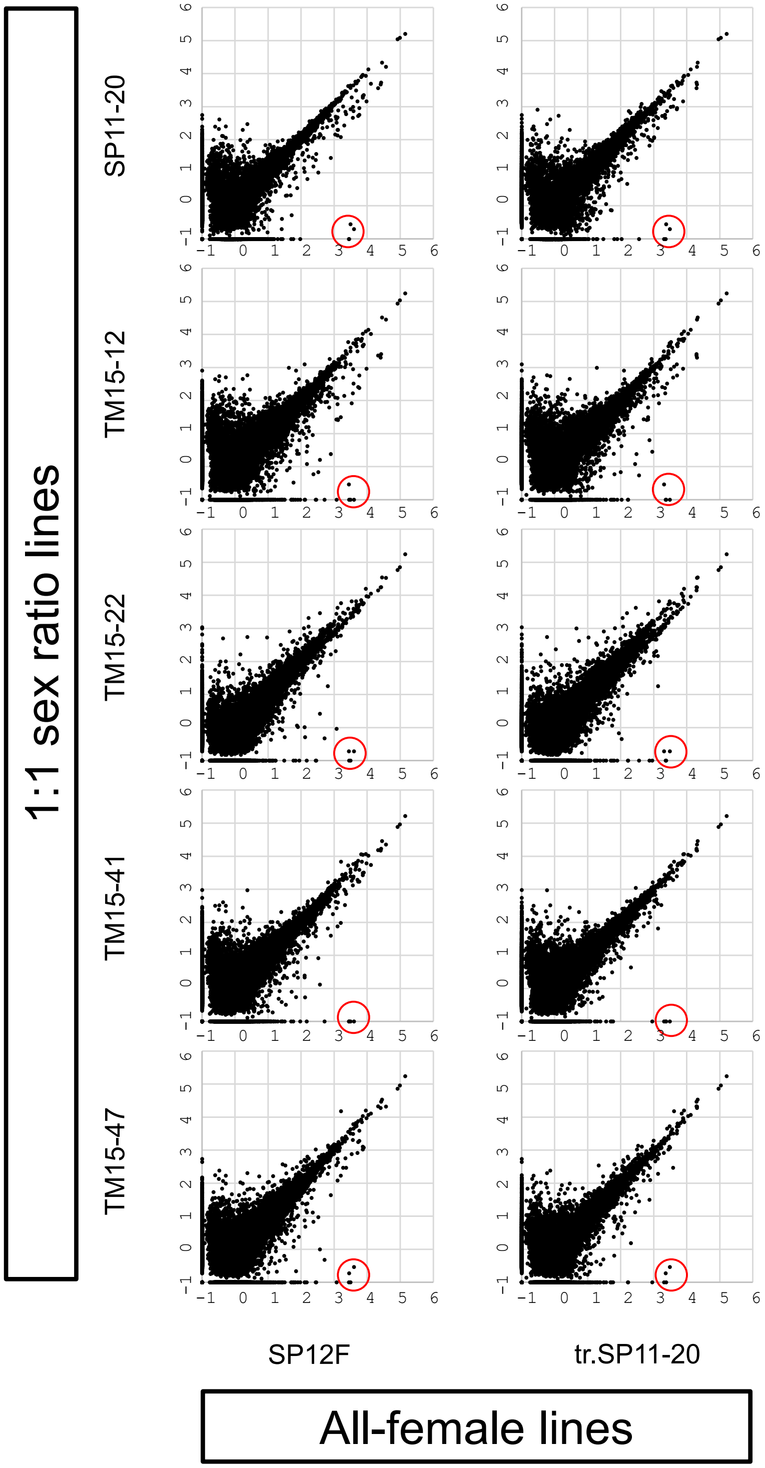

Supplementary Figure 3. Comparison of TPM between all-female matrilines (SP12F or tr.SP11-20) and normal sex ratio lines (SP11-20, TM15-12, TM15-22, TM15-41 or TM15-47). Red circles indicate the four contigs that exclusively present in all-female matrilines. The $\log_{10}$(TPM+0.1) was plotted for each contig. Source data are deposited in https://doi.org/10.6084/m9.figshare.22047350.v1.

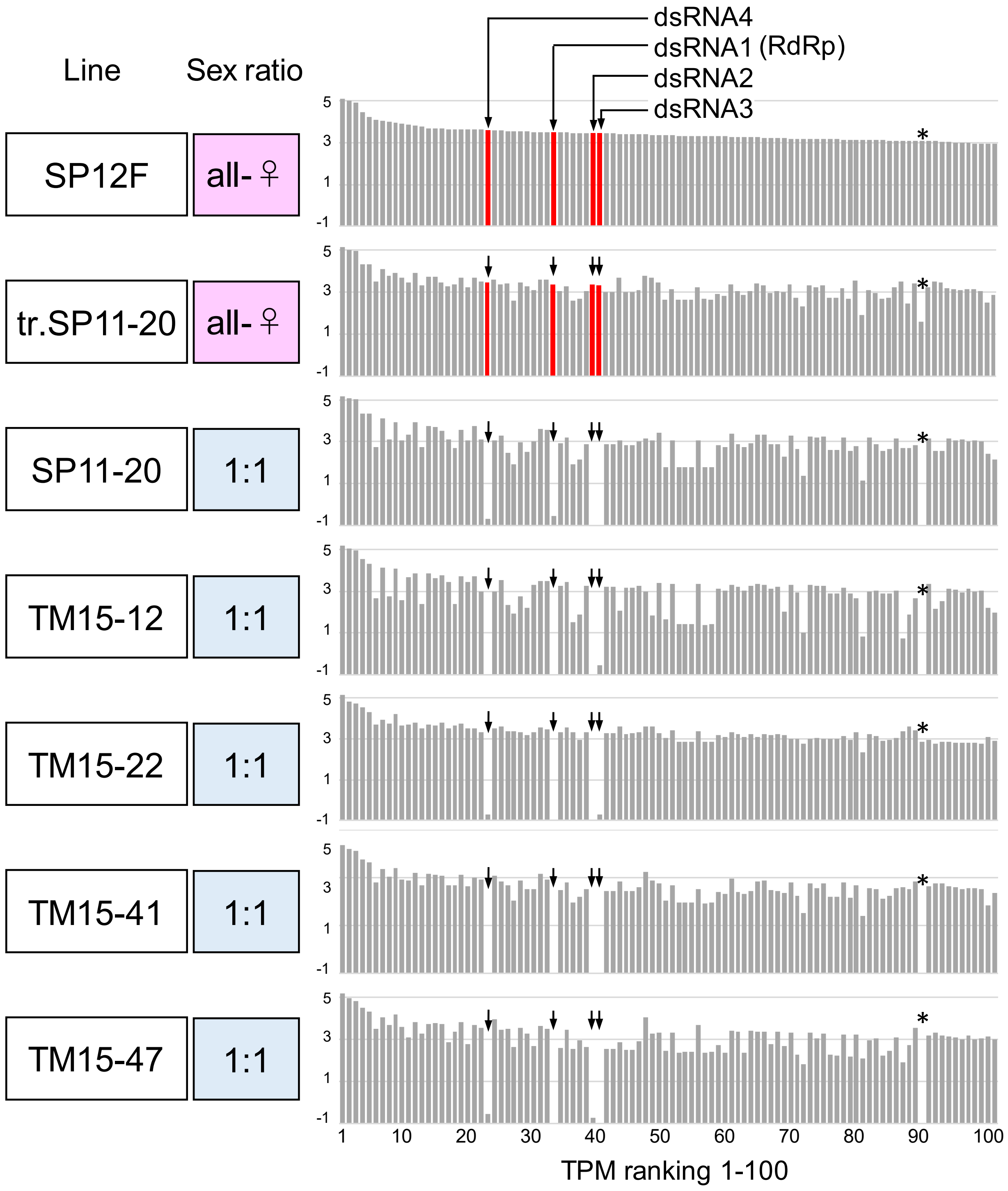

Supplementary Figure 4. One-hundred contigs that showed highest level of expression in SP12F. Four contigs, indicated by arrows, were exclusively found in SP12F and tr.SP11-20 (red bars). The expression level is shown with $\log_{10}$(TPM+0.1). The contig indicated by asterisks is *Gluconacetobacter* sp., a gut symbiont of *Drosophila*. Source data are deposited in https://doi.org/10.6084/m9.figshare.22047350.v1.

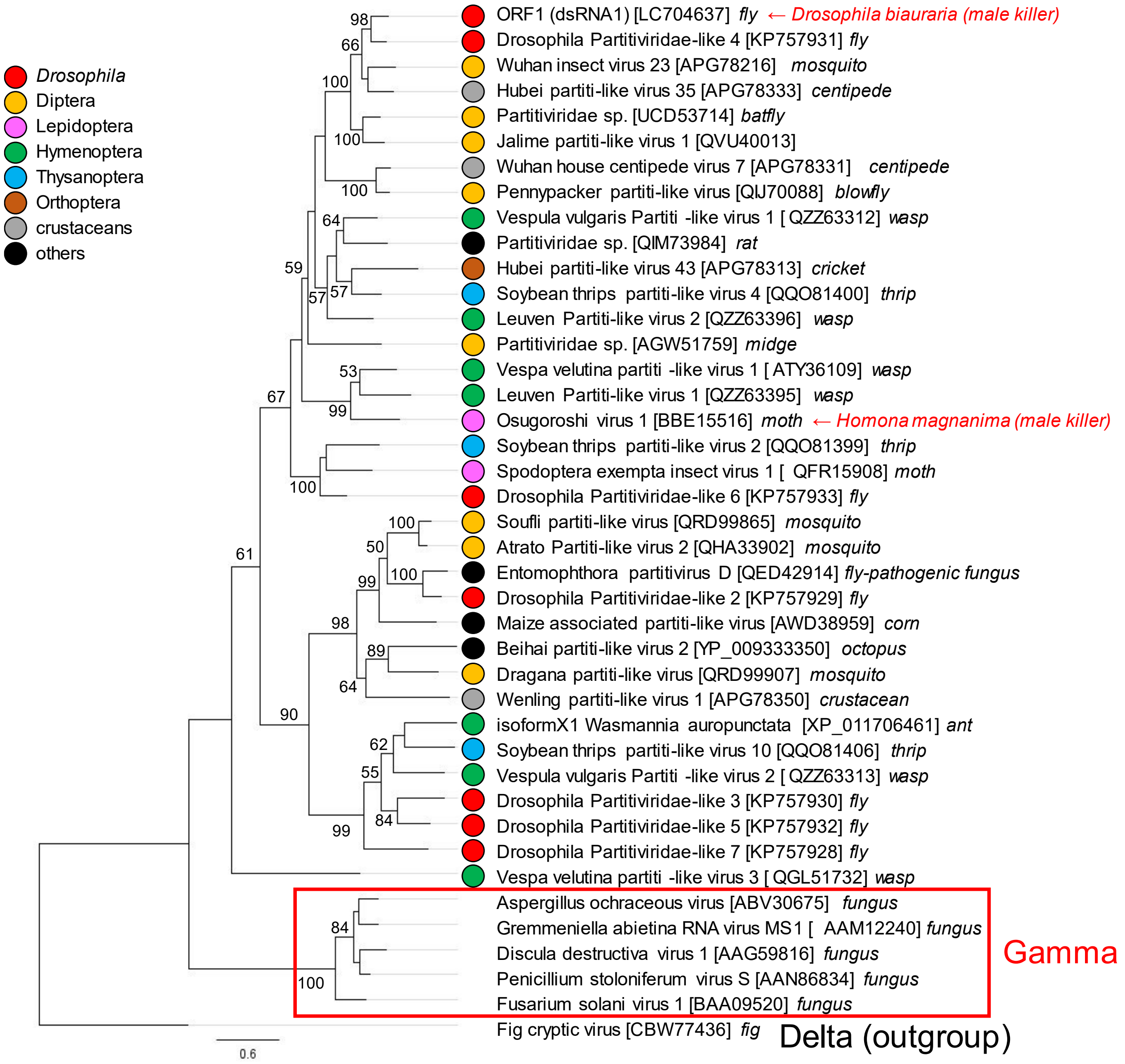

Supplementary Figure 5. Phylogenetic position of ORF1 encoded by dsRNA1 among closely related partiti-like viruses. Maximum likelihood tree based on amino acid sequences of RdRp is shown. Gammapatitivirus are framed with red. Fig cryptic virus (Deltapartitivirus) is used as outgroup. Source data are provided in Supplementary Data 2.

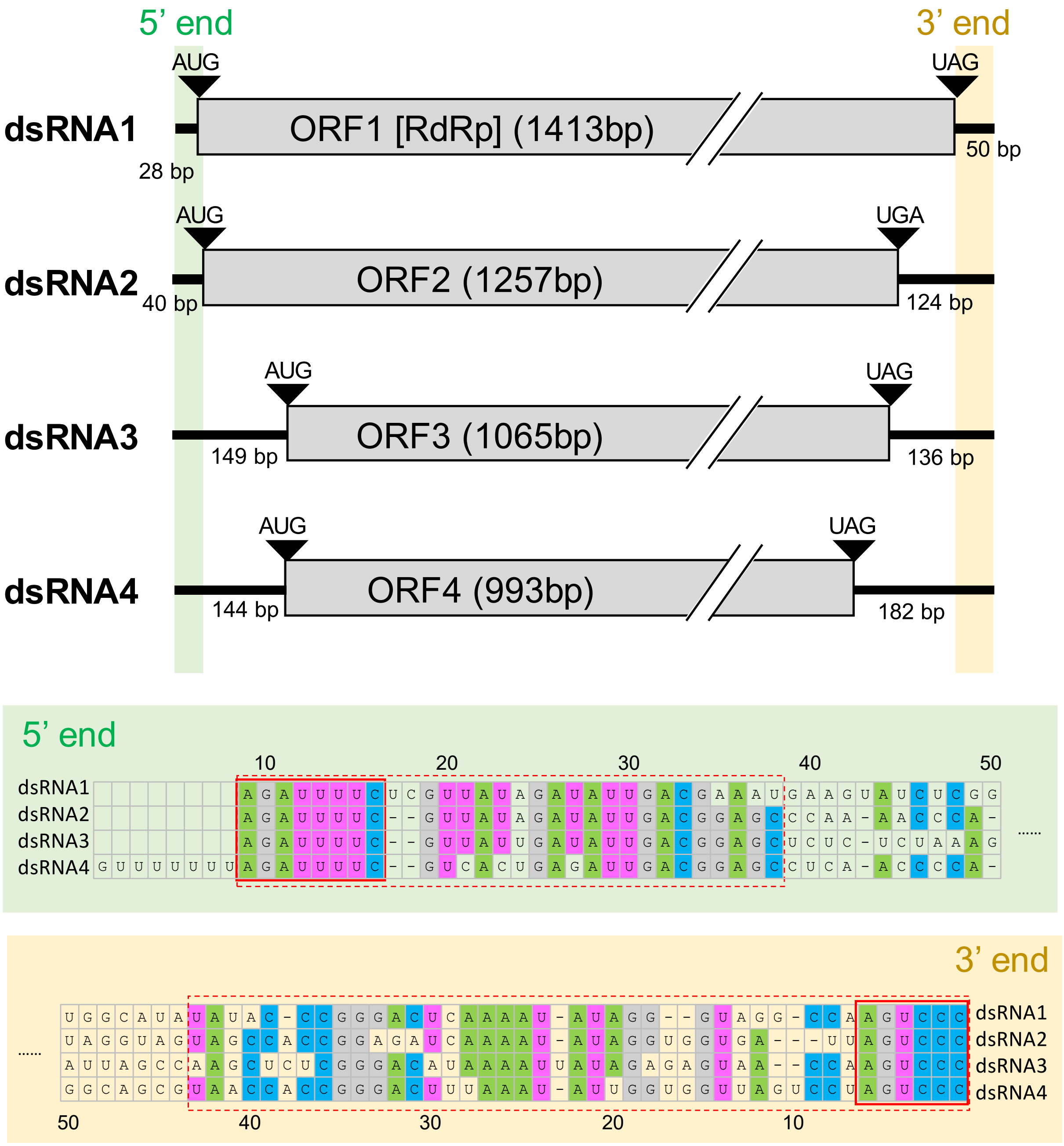

Supplementary Figure 6. Nucleotide sequences at the 5'-ends and 3'-ends of the four double-stranded RNA sequences (dsRNA1–dsRNA4). (Upper) Black bars represent untranslated regions (UTRs). Gray boxes represent open reading frames (ORFs). Triangles represent start or stop codons. (Bottom) Partial nucleotide sequences of 5'-end and 3'-end are shown. Fixed and similar sequences are surrounded by red lines and red dotted lines, respectively. Source data are deposited in DDBJ accession numbers LC704637–LC704640.

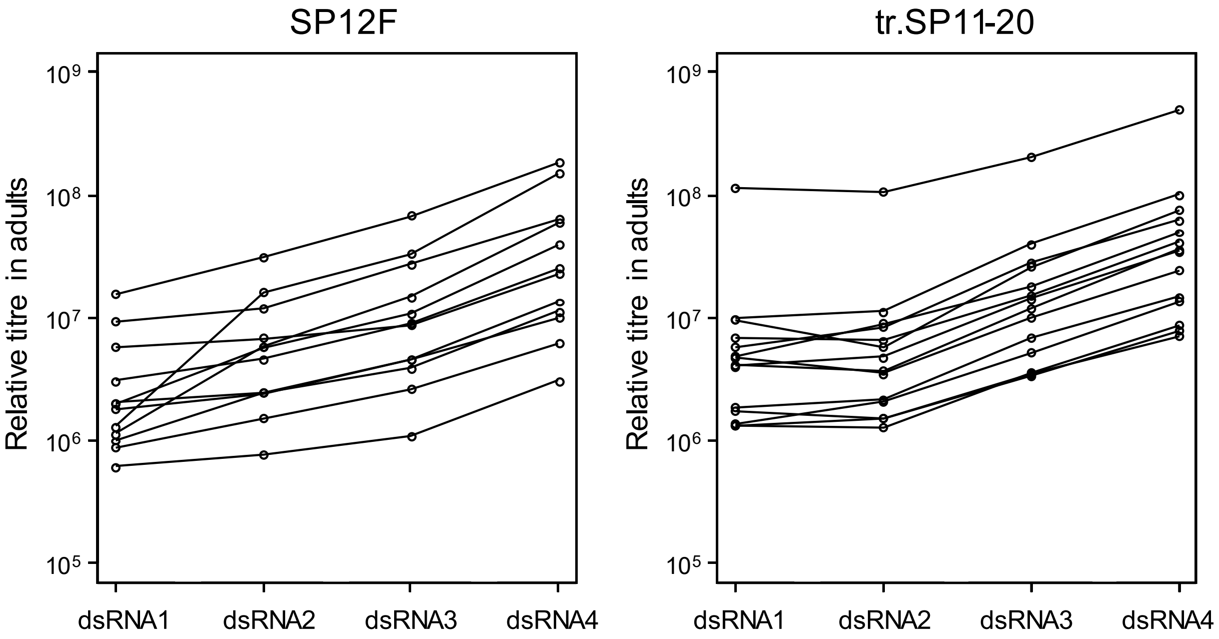

Supplementary Figure 7. Relative titres of dsRNA1–dsRNA4 in adults of SP12F (left) and tr.SP11-20 (right). Each plot represents an individual. The plots from the same individuals are connected by lines. RNA titres of dsRNA1–dsRNA4 relative to those of ribosomal protein 49 (rp49) gene are estimated by delta-CT method. Source data are provided in Supplementary Data 3.

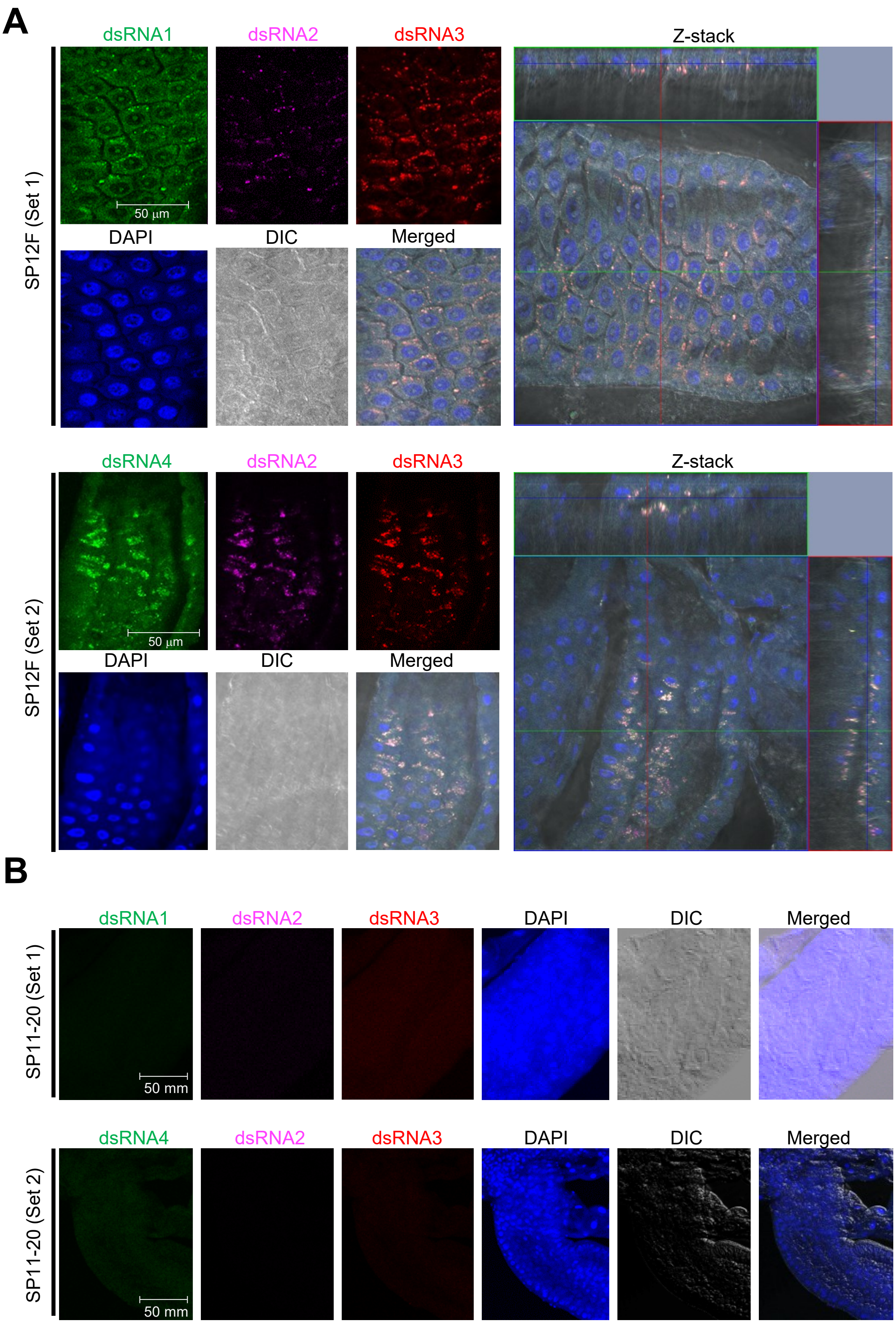
A
dsRNA1
dsRNA2
dsRNA3
Z-stack
SP12F (Set 1)
50 µm
DAPI
DIC
Merged
dsRNA4
dsRNA2
dsRNA3
Z-stack
SP12F (Set 2)
50 µm
DAPI
DIC
Merged
B
dsRNA1
dsRNA2
dsRNA3
DAPI
DIC
Merged
SP11-20 (Set 1)
50 mm
dsRNA4
dsRNA2
dsRNA3
DAPI
DIC
Merged
SP11-20 (Set 2)
50 mm

Supplementary Figure 8. FISH images in *D. biauraria*. (A) A midgut tissue of a female adult of all-female matriline SP12F. SP12F (Set 1, n = 9): *in situ* hybridization using the fluorescent probes of dsRNA1, dsRNA2, and dsRNA3 (green, magenta, and red, respectively), and counterstained with DAPI (blue). DIC: differential interference contrast images. SP12F (Set 2, n =9): *in situ* hybridization using the fluorescent probes of dsRNA4, dsRNA2, and dsRNA3 (green, magenta, and red, respectively) and counterstained with DAPI (blue). Z-stack: three-dimensional reconstruction using Z-stack acquisitions. Part of these photos are shown in Fig. 2D. (B) A midgut tissue of a female adult of the normal sex ratio line SP11-20. SP11-20 (Set 1, n = 6): *in situ* hybridization using the fluorescent probes of dsRNA1, dsRNA2, and dsRNA3 (green, magenta, and red, respectively), and counterstained with DAPI (blue). SP11-20 (Set 2, n = 6): *in situ* hybridization using the fluorescent probes of dsRNA4, dsRNA2, and dsRNA3 (green, magenta, and red, respectively), and counterstained with DAPI (blue).

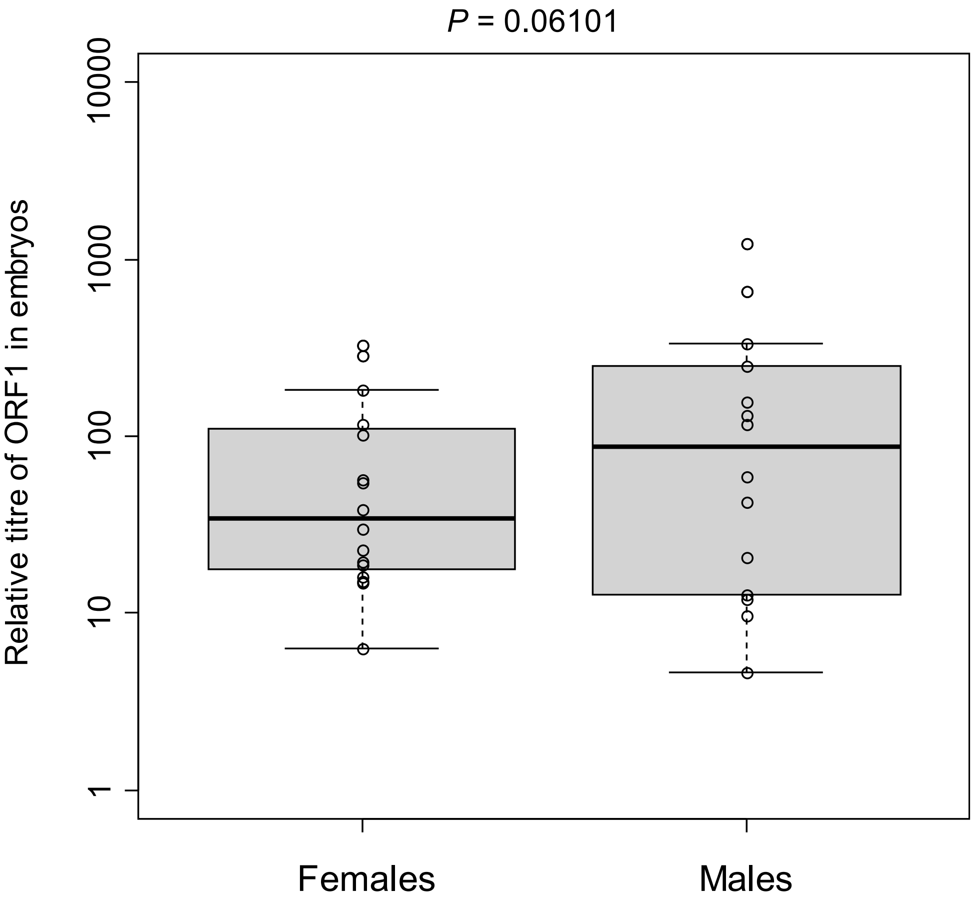

Supplementary Figure 9. Relative titres of dsRNA1 in embryos of DbMKPV1-infected *D. biauraria* (SP12F). From a single embryo oviposited within 24 h under 20 °C, DNA and RNA were extracted separately. According to the sexing based on Y chromosome-specific PCR performed on 30 embryos, 16 and 14 embryos were considered females and males, respectively. For these embryos, RNA titres of dsRNA1 relative to those of rp49 gene were estimated by qPCR using delta-CT method. Each plot represents a single embryo. Box plots represent the median, the first quartiles and third quartiles with whiskers drawn within the 1.5 IQR value. For comparison of dsRNA1 titres between females and males, statistical test was performed based on generalized linear model. P-value of 0.06101 was obtained based on negative binomial distribution, which was selected by the Akaike information criterion from normal, gamma and negative binomial distribution. Points outside the whiskers are outliers. Source data are provided in Supplementary Data 6.

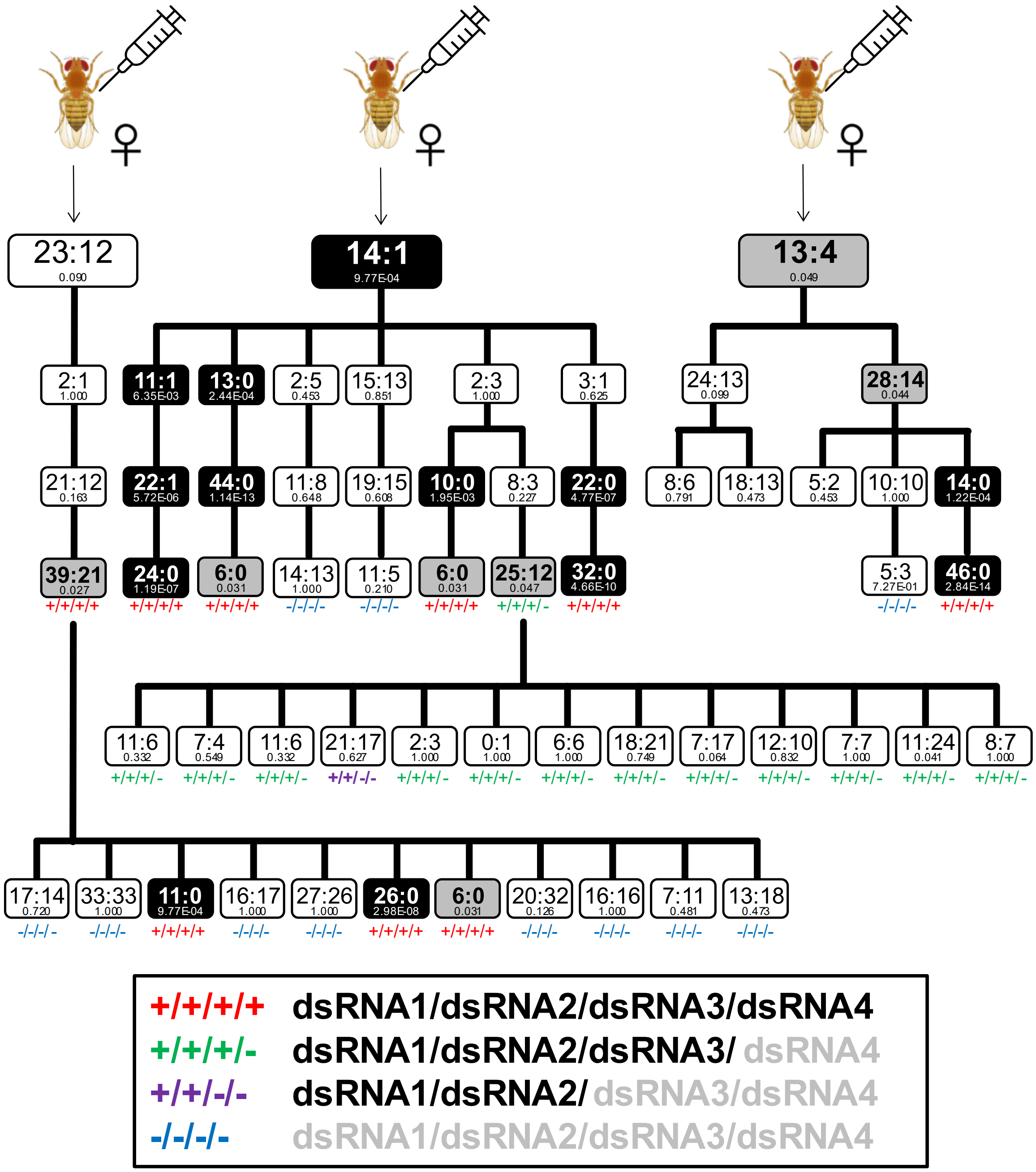

Supplementary Figure 10. Pedigree of *D. biauraria* (line SP11-20) following injection of filter-sterilized homogenate of SP12F. Homogenate of SP12F was passed through a 0.22 µm-filter and was injected into three adult females of SP11-20. Each box represents a brood with large letters showing numbers of emerged adults (females : males) and small letters below showing P-values of binomial test (two-sided). Black boxes represent broods with strongly female-biased sex ratio ($P < 0.01$). Gray boxes represent broods with mildly female-biased sex ratio ($P < 0.05$). White boxes represent broods with nonbiased or male-biased sex ratio. Shown below each brood is the infection status of the mother (Presence or absence of dsRNA1, dsRNA2, dsRNA3 and dsRNA4 is indicated as + or -).

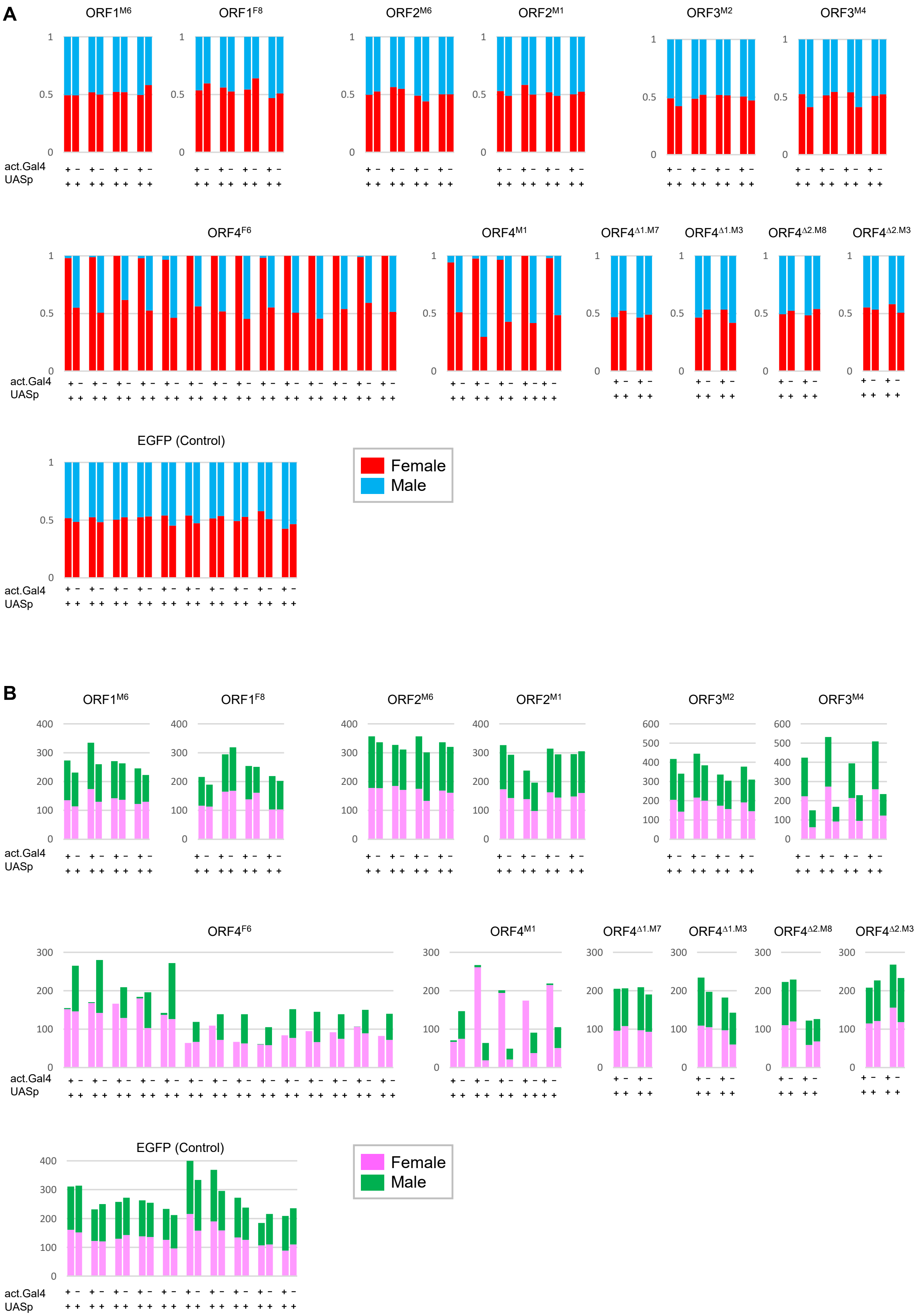
A
ORF1^{M6}
ORF1^{F8}
ORF2^{M6}
ORF2^{M1}
ORF3^{M2}
ORF3^{M4}
ORF4^{F6}
ORF4^{M1}
ORF4$^{\Delta1.M7}$
ORF4$^{\Delta1.M3}$
ORF4$^{\Delta2.M8}$
ORF4$^{\Delta2.M3}$
EGFP (Control)
act.Gal4
UASp
Female
Male
B
ORF1^{M6}
ORF1^{F8}
ORF2^{M6}
ORF2^{M1}
ORF3^{M2}
ORF3^{M4}
ORF4^{F6}
ORF4^{M1}
ORF4$^{\Delta1.M7}$
ORF4$^{\Delta1.M3}$
ORF4$^{\Delta2.M8}$
ORF4$^{\Delta2.M3}$
EGFP (Control)
act.Gal4
UASp
Female
Male

Supplementary Figure 11. Effects of ORF overexpression on the sex ratio of emerged adults in *Drosophila melanogaster* (using actin ubiquitous driver). (A) Proportions of males and females. (B) Number of males and females. ORF1, ORF2, ORF3, ORF4, two frameshifted ORF4 (1 bp and 2 bp deletions), and EGFP were ectopically expressed. For each brood, overexpressed individuals (act.Gal4-positive; UASp-positive) and non-overexpressed control (act.Gal4-negative; UASp-positive) are shown in a pair. Source data are provided in Supplementary Data 4.

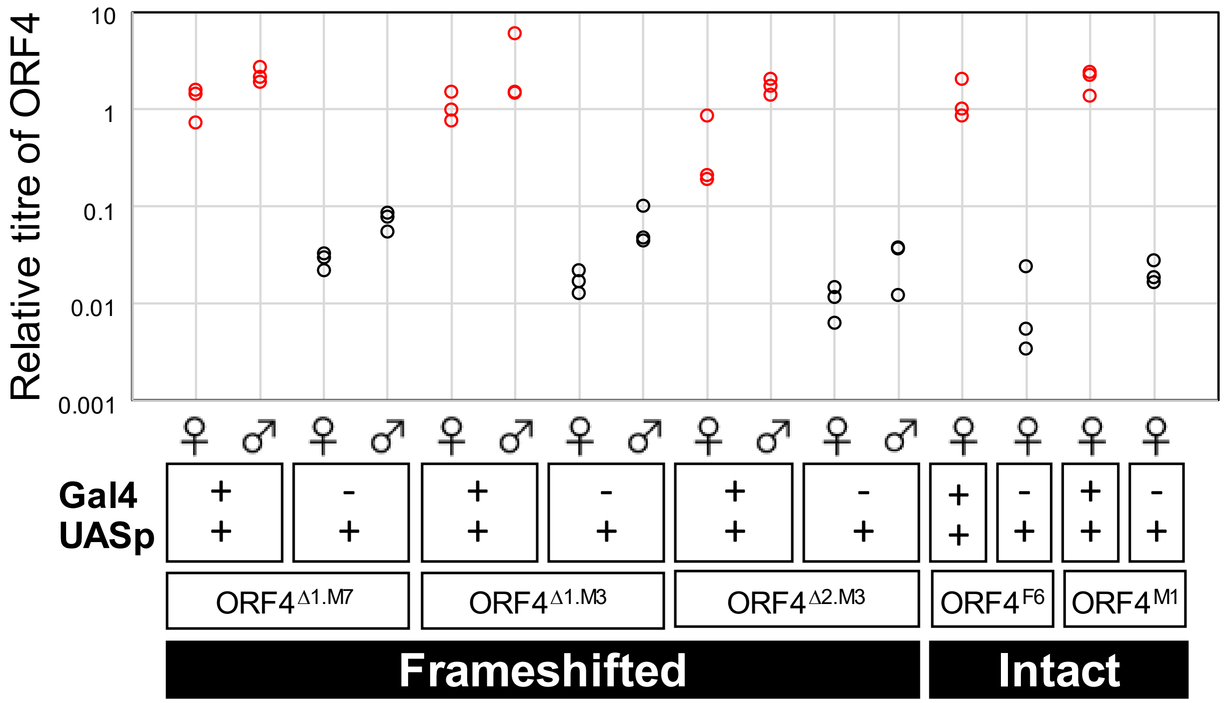

Supplementary Figure 12. Relative level of ORF4 expression in transgenic *D. melanogaster* estimated by quantitative real-time PCR. Relative copy numbers of ORF4 per rp49 copy were estimated by delta-CT method. The value of a female wherein intact ORF4^{F6} was overexpressed was set to 1. Red circles indicate overexpressed samples (Gal4 driver and UASp) and black circles indicate non-overexpressed samples (UASp only). Three individuals were examined for each cohort. Source data are provided in Supplementary Data 5.

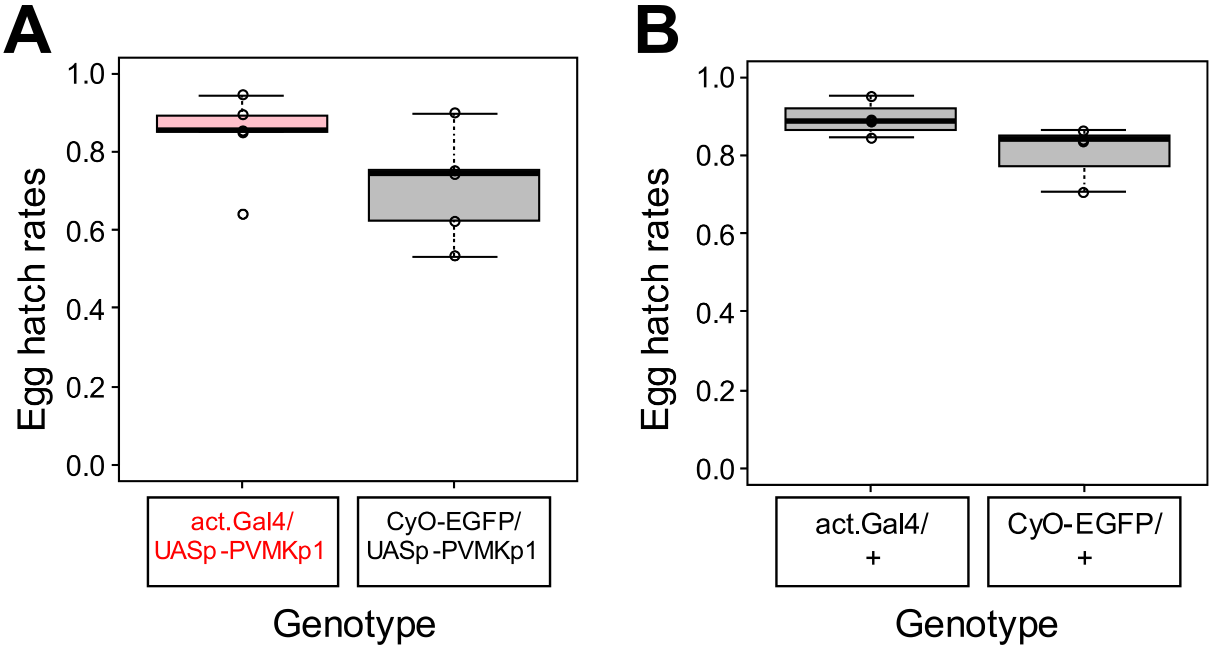

Supplementary Figure 13. Effects of PVMKp1 (ORF4) overexpression on the egg hatch rates of *D. melanogaster*. (A) Egg hatch rates of offspring produced by act.Gal4/CyO-EGFP (mothers) and UASp-PVMKp1/UASp-PVMKp1 (fathers). n = 400, 364, 449, 305 and 399 eggs were examined over 5 independent experiments. (B) Egg hatch rates of offspring produced by Act.Gal4/CyO-EGFP (mothers) and +/+ (OR-NIG) (fathers). n = 198, 518, 269 and 229 eggs were examined over 4 independent experiments. Box plots represent the median, the first quartiles and third quartiles with whiskers drawn within the 1.5 IQR value. Points outside the whiskers are outliers. Source data are provided in Supplementary Data 7.

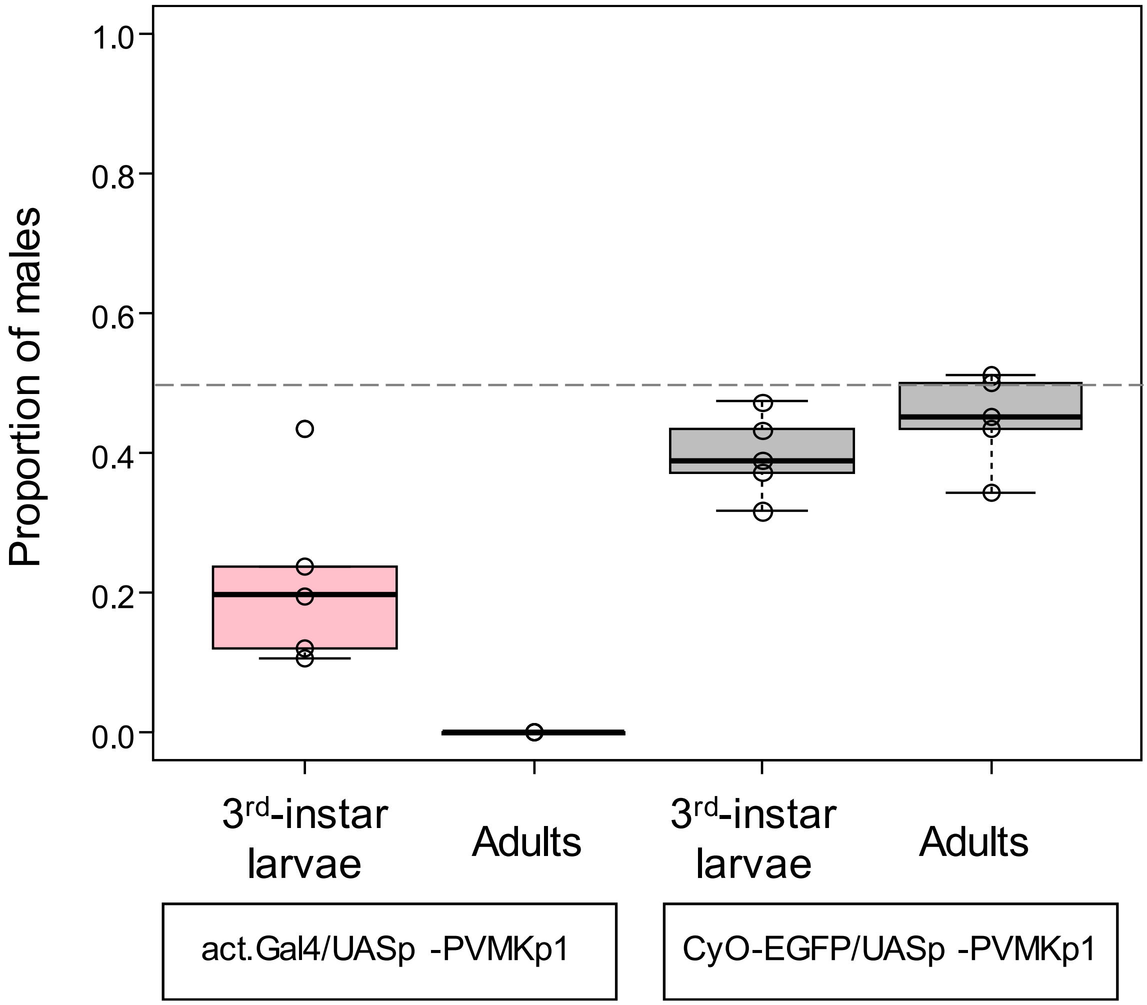

Supplementary Figure 14. Effects of PVMKp1 (ORF4) overexpression on the sex ratio of the third-instar larvae and adults of *D. melanogaster*. Offspring produced by mating between act.Gal4/CyO-EGFP females and UASp-PVMKp1/UASp-PVMKp1 males were sampled at the third-instar larval stage and adult stage. After genotyping by the presence/absence of EGFP signals, sexing was made based on sex-specific morphological characters. Over 5 independent experiments, n = 212, 210, 156, 151 and 57 3rd-instar larvae and n = 163, 149, 145, 177 and 160 adults were examined. Box plots represent the median, the first quartiles and third quartiles with whiskers drawn within the 1.5 IQR value. Points outside the whiskers are outliers. Source data are provided in Supplementary Data 7.

Supplementary Table 1. Primers and probes used in this study.

Name	Target	Sequence	Purpose
1_front_0253R	Partitivirus, dsRNA1	5'- ACATTGGAATTCGGTCCGGT -3'	RACE
1_front_0243R	Partitivirus, dsRNA1	5'- TCGGTCCGGTAGCTGAAAAG -3'	RACE
1_back_1175F	Partitivirus, dsRNA1	5'- TTTGGCCTGAGCGACATGAT -3'	RACE
1_back_1104F	Partitivirus, dsRNA1	5'- AACGTTCCTTGGCTACAGCA -3'	RACE
2_front_0273R	Partitivirus, dsRNA2	5'- AACCGGTTGTGCGTTGATTG -3'	RACE
2_front_0346R	Partitivirus, dsRNA2	5'- TGGTGCAAGTCCGGCTATAC -3'	RACE
2_back_1049F	Partitivirus, dsRNA2	5'- CAACCCCCGGTGAAGTAGTC -3'	RACE
2_back_1123F	Partitivirus, dsRNA2	5'- CCGCAGCCAATCAAAACCAT -3'	RACE
3_front_0301R	Partitivirus, dsRNA3	5'- ACCACGCCAGACTCTTAAGC -3'	RACE
3_front_0242R	Partitivirus, dsRNA3	5'- CGATGAACGGCCATTTGCTT -3'	RACE
3_back_1086F	Partitivirus, dsRNA3	5'- TGGACTCGCTAGGACTATGCT -3'	RACE
3_back_1138F	Partitivirus, dsRNA3	5'- AAGTTGCTCGCTGAGTCGAA -3'	RACE
4_front_0239R	Partitivirus, dsRNA4	5'- AGCGCTCAAGTAACGAGAACT -3'	RACE
4_front_0286R	Partitivirus, dsRNA4	5'- AGCACTACGAATAGGACGTGG -3'	RACE
4_back_1013F	Partitivirus, dsRNA4	5'- CGGGCCAACAAAATCTTGGT -3'	RACE
4_back_0934F	Partitivirus, dsRNA4	5'- TGCATTGAGTCTAAGCCGGA -3'	RACE
ORF1_1F	Partitivirus, dsRNA1	5'- CACCATGAAGTATCTCGGAAAGGCATCTGG -3'	Vector construction
ORF1_1410R	Partitivirus, dsRNA1	5'- GCAGAAGCCAAGCCGTCGCATAAAC -3'	Vector construction
ORF2_1F	Partitivirus, dsRNA2	5'- CACCATGGAGATCAAGGAAGAATCTGATACTGG -3'	Vector construction
ORF2_1254R	Partitivirus, dsRNA2	5'- TTTTCGAGGTGCGTCACAAAGGTTC -3'	Vector construction
ORF3_1F	Partitivirus, dsRNA3	5'- CACCATGCCCAGACCAAGAAGTACTCCG -3'	Vector construction
ORF3_1062R	Partitivirus, dsRNA3	5'- AACTTCCAACTTCTCAAAATCCATCACC -3'	Vector construction
ORF4_1F	Partitivirus, dsRNA4	5'- CACCATGGCGCATGCTCAAGCAAGTAATG -3'	Vector construction

ORF4_990R	Partitivirus, dsRNA4	5'- AGGCTCGCAAAACTGCCTACTAACC -3'	Vector construction
ORF4 (-1)_1F	Partitivirus, dsRNA4	5'- CACCATGCGCATGCTCAAGCAAGTAATG -3'	Vector construction
ORF4 (-2)_1F	Partitivirus, dsRNA4	5'- CACCATGGCATGCTCAAGCAAGTAATG -3'	Vector construction
m3529_p83, AF488-labeled	Partitivirus, dsRNA1	5'- TGATAATCGCCAGTCAGAGGAGCCA -3'	FISH
m3529_p192, AF488-labeled	Partitivirus, dsRNA1	5'- AAGTGCGTCGTATCGCTTCGTCAAAC -3'	FISH
m10641_p226, AF647-labeled	Partitivirus, dsRNA2	5'- CAACCGGTTGTGCGTTGATTGGTAC -3'	FISH
m10641_p946, AF647-labeled	Partitivirus, dsRNA2	5'- CGTGATTCGAACGTAGAGACAATCCG -3'	FISH
m5059_p465, AF555-labeled	Partitivirus, dsRNA3	5'- GTCCAACTAGCGTGCTATCAATTGAG -3'	FISH
m5059_p546, AF555-labeled	Partitivirus, dsRNA3	5'- GTCCCATCTTCGCACGAGGATTCTTT -3'	FISH
m3342_p224, AF488-labeled	Partitivirus, dsRNA4	5'- CGTATTTCACGCATTACGTGAGCGG -3'	FISH
m3342_p325, AF488-labeled	Partitivirus, dsRNA4	5'- CTGGCGAACAGTTTAACTGCCTTCGA -3'	FISH
ORF4_q_F1	Partitivirus, dsRNA4	5'- GCTTACAGAGCAACTAACATCGTCTTTG -3'	qPCR
ORF4_q_R1	Partitivirus, dsRNA4	5'- CCTATGGTGCCGTATTTCACGC -3'	qPCR
Dm_rp49_q_F1	*D. melanogaster*, rp49	5'- GCACTTCATCCGCCACCAGTC -3'	qPCR
Dm_rp49_q_R1	*D. melanogaster*, rp49	5'- GCACTCTGTTGTCGATACCCTTGG -3'	qPCR
DbY_c52202_F2	*D. biauraria*, Y-linked	5'- ACCGAGCGCGAAATCATAAAACCAGCATC -3'	Sexing
DbY_c52202_R2	*D. biauraria*, Y-linked	5'- CTCATATCACTTCATGTATCCCACACTTTTAACAG -3'	Sexing
Db-actin5C-68-F	*D. biauraria*, actin5C	5'- GGCCATCCAGGCCGTGCTCTC -3'	Sexing
Db-actin5C-68-R	*D. biauraria*, actin5C	5'- GCGCTCGGCAGTGGTGGTGAAG -3'	Sexing

Supplementary Table 2. Homology searches (blastp and blastn) for the four double-stranded RNA sequences.

Query	blastp search			blastn Search		
	Description	Accession #	E-value	Description	Accession #	E-value
ORF1 (RdRp)	RdRp [Hubei partiti-like virus 35][1] (host: house centipede)	APG78333	0	*Drosophila*-associated Partitiviridae-like 4 clone[2]	KP757931	3×10^{-174}
	RdRp [Wuhan insect virus 23][1] (host: mosquitoes)	APG78216	0			
ORF2	hypothetical protein [Wuhan insect virus 23][1] (host: mosquitoes)	YP_009329883	4×10^{-92}	Small RNA [DmelPosVir_26][3]	KP757962	4×10^{-25}
ORF3	hypothetical protein [*Microbacterium arborescens*]	WP_162815286	7×10^{-11}	Small RNA [DmelPosVir_36][3]	KP757972	2×10^{-98}
ORF4	No hits	-	-	No hits	-	-

[1]Partitiviridae-like virus (Shi *et al.*, 2016).
[2]Partitiviridae-like virus (Webster *et al.*, 2015).
[3]Putative viral sequences (Webster *et al.*, 2015).

Supplementary References

1. Shi, M. et al. Redefining the invertebrate RNA virosphere. *Nature* **540**, 539–543 (2016).
2. Webster, C. L. et al. The discovery, distribution, and evolution of viruses associated with *Drosophila melanogaster. PLoS Biol.* **13**, e1002210 (2015).
