# Supplementary material for: A male-killing gene encoded by a symbiotic virus of Drosophila
Source: Nat Commun. 2023 Mar 13;14:1357. doi: 10.1038/s41467-023-37145-0 (PMC10011393; doi:10.1038/s41467-023-37145-0)
Supplement: Supplementary file 3 — Reporting Summary [file 41467_2023_37145_MOESM3_ESM.pdf]

nature portfolio

Corresponding author(s): *Double-blind peer review submissions: write DBPR and your manuscript number here instead of author names.*

Last updated by author(s): *YYYY-MM-DD*

Reporting Summary

Nature Portfolio wishes to improve the reproducibility of the work that we publish. This form provides structure for consistency and transparency in reporting. For further information on Nature Portfolio policies, see our Editorial Policies and the Editorial Policy Checklist.

Statistics

For all statistical analyses, confirm that the following items are present in the figure legend, table legend, main text, or Methods section.

n/a	Confirmed	
☐	☒	The exact sample size (*n*) for each experimental group/condition, given as a discrete number and unit of measurement
☐	☒	A statement on whether measurements were taken from distinct samples or whether the same sample was measured repeatedly
☐	☒	The statistical test(s) used AND whether they are one- or two-sided<br>*Only common tests should be described solely by name; describe more complex techniques in the Methods section.*
☐	☒	A description of all covariates tested
☐	☒	A description of any assumptions or corrections, such as tests of normality and adjustment for multiple comparisons
☐	☒	A full description of the statistical parameters including central tendency (e.g. means) or other basic estimates (e.g. regression coefficient) AND variation (e.g. standard deviation) or associated estimates of uncertainty (e.g. confidence intervals)
☐	☒	For null hypothesis testing, the test statistic (e.g. *F*, *t*, *r*) with confidence intervals, effect sizes, degrees of freedom and *P* value noted<br>*Give P values as exact values whenever suitable.*
☐	☒	For Bayesian analysis, information on the choice of priors and Markov chain Monte Carlo settings
☒	☐	For hierarchical and complex designs, identification of the appropriate level for tests and full reporting of outcomes
☒	☐	Estimates of effect sizes (e.g. Cohen's *d*, Pearson's *r*), indicating how they were calculated

Our web collection on statistics for biologists contains articles on many of the points above.

Software and code

Policy information about availability of computer code

Data collection	No software was used.
Data analysis	Trimmomatic (version 0.36) for RNA-seq analysis<br>Trinity (version 2.2.0) for RNA-seq analysis<br>TransDecoder (version 3.0.1) for RNA-seq analysis<br>HMMER (version 3.1b2) for RNA-seq analysis<br>Bowtie2 (version 2.2.6) for RNA-seq analysis<br>RSEM (version 1.2.31) for RNA-seq analysis<br>MAFFT (version 7) for phylogenetic analysis<br>trimAL (version 1.3) for phylogenetic analysis<br>raxmlGUI 2.0 (version 2.0.7) for phylogenetic analysis<br>ModelTest-NG (version 0.1.7) for phylogenetic analysis<br>Stellaris Probe Designer (version 4.2) for probe design<br>ProbeCheck for checking probes<br>BLASTN (version 2.6.1) for blast search<br>ZEN2009 (Carl Zeiss) for image analysis<br>EBImage package for R (version 4.5.22) for imaging analysis<br>R (version 4.1.2) for statistical analysis, data plot and imaging analysis<br>Fiji ImageJ version 1.51r for imaging analysis

For manuscripts utilizing custom algorithms or software that are central to the research but not yet described in published literature, software must be made available to editors and reviewers. We strongly encourage code deposition in a community repository (e.g. GitHub). See the Nature Portfolio guidelines for submitting code & software for further information.

Data

Policy information about availability of data

All manuscripts must include a data availability statement. This statement should provide the following information, where applicable:
- Accession codes, unique identifiers, or web links for publicly available datasets
- A description of any restrictions on data availability
- For clinical datasets or third party data, please ensure that the statement adheres to our policy

RNA-seq raw data generated in this study have been deposited to DDBJ Sequence Read Archive (DRA) under the accession number DRA011109. RNA-seq contig data are deposited in https://doi.org/10.6084/m9.figshare.22047350.v1. All the other data are available in the main text or the supplementary materials with source data provided as a Source Data file.

Human research participants

Policy information about studies involving human research participants and Sex and Gender in Research.

Reporting on sex and gender	Not applicable.
Population characteristics	Not applicable.
Recruitment	Not applicable.
Ethics oversight	Not applicable.

Note that full information on the approval of the study protocol must also be provided in the manuscript.

Field-specific reporting

Please select the one below that is the best fit for your research. If you are not sure, read the appropriate sections before making your selection.

[x] Life sciences [ ] Behavioural & social sciences [ ] Ecological, evolutionary & environmental sciences

For a reference copy of the document with all sections, see nature.com/documents/nr-reporting-summary-flat.pdf

Life sciences study design

All studies must disclose on these points even when the disclosure is negative.

Sample size	For egg hatch rates , 200 gravid adult females of 7-14 days after eclosion were allowed to lay eggs overnight on grape juice agar plate. All the laid eggs (if > 100) were used to measure egg hatch rates. For sex ratios, all the flies emerged by 18th day after the start of egg oviposition were counted (Harumoto and Lemaitre, 2018 Nature 557: 252-255).
Data exclusions	No data were excluded.
Replication	All attempts to repeat the experiment were successful.
Randomization	For each replicates, we randomly chose a fixed number for embryos, larvae or adults of D. biauraria or D. melanogaster.
Blinding	For sex ratio analysis, we systematically aligned the vials and counted the number of males and females in order. Each vial was labeled but we

Reporting for specific materials, systems and methods

We require information from authors about some types of materials, experimental systems and methods used in many studies. Here, indicate whether each material, system or method listed is relevant to your study. If you are not sure if a list item applies to your research, read the appropriate section before selecting a response.

Materials & experimental systems

n/a	Involved in the study	
☐	☒	Antibodies
☒	☐	Eukaryotic cell lines
☒	☐	Palaeontology and archaeology
☒	☐	Animals and other organisms
☒	☐	Clinical data
☒	☐	Dual use research of concern

Methods

n/a	Involved in the study	
☒	☐	ChIP-seq
☒	☐	Flow cytometry
☒	☐	MRI-based neuroimaging

Antibodies

Antibodies used

rabbit anti-acetyl-histone H4 lysine 16 (H4K16ac) (Sigma-Aldrich, 07-329)
mouse anti-phospho-histone H2Av (pH2Av) (DSHB, UNC93-5.2.1)
donkey anti-mouse IgG (H+L) highly cross-adsorbed secondary antibody, Alexa Fluor Plus 555 (Thermo Fisher Scientific, A32773)
donkey anti-rabbit IgG (H+L) highly cross-adsorbed secondary antibody, Alexa Fluor Plus 647 (Thermo Fisher Scientific, A32795)

Validation

H4K16ac: Lucchesi, J.C., and Kuroda, M.I. (2015). Dosage compensation in Drosophila. Cold Spring Harb. Perspect. Biol. 7, a019398
pH2Av: DSHB, UNC93-5.2.1 (Drosophila). Madigan, J.P., Chotkowski, H.L., and Glaser, R.L. (2002). DNA double-strand break-induced phosphorylation of Drosophila histone variant H2Av helps prevent radiation-induced apoptosis. Nucleic Acids Res. 30, 3698-3705.
