# Supplementary material for: A male-killing gene encoded by a symbiotic virus of Drosophila
Source: Nat Commun. 2023 Mar 13;14:1357. doi: 10.1038/s41467-023-37145-0 (PMC10011393; doi:10.1038/s41467-023-37145-0)
Supplement: Supplementary file 4 — Description of additional supplementary files [file 41467_2023_37145_MOESM4_ESM.docx]

**Description of Additional Supplementary Files**

Supplementary Data 1

Description: Count data of *D. biauraria*. The "hatch rate" sheet shows the data of egg hatch rates used in Fig. 1A and Supplementary Fig. 1. The "sexing" sheet shows the number of males and females used in Fig. 1B.

Supplementary Data 2

Description: Amino acid sequences of RdRp and host information of the viruses used for phylogenetic analysis. The "107OTU" sheet shows the 107 amino acid sequences used for phylogenetic tree reconstruction in Fig. 2C. The "41OTU" sheet shows the 41 amino acid sequences used for phylogenetic tree reconstruction in Supplementary Fig. 5.

Supplementary Data 3

Description: Titres of dsRNA1-dsRNA4 in adult *D. biauraria*. The "sheet 1" shows the qPCR data on dsRNA1-dsRNA4 in adults of SP12F and tr.SP11-20 (Supplementary Fig. 7).

Supplementary Data 4

Description: Count data of *D. melanogaster* adults. The "act.Gal4" sheet shows the effect on sex ratio of overexpression using actin driver (Fig. 3B; Supplementary Fig. 11). The "nos.Gal4" sheet shows the effect on sex ratio of overexpression using nanos driver (Fig. 3C).

Supplementary Data 5

Description: qPCR data on overexpressed *D. melanogaster*. The "qPCR" sheet shows the qPCR data on overexpressed *D. melanogaster* (Supplementary Fig. 12).

Supplementary Data 6

Description: Virus titres in male and female embryos of *D. biauraria*. The "sheet 1" shows the qPCR data on dsRNA1 in male and female embryos of SP12F (Supplementary Fig. 9).

Supplementary Data 7

Description: Survivorship of overexpressed *D. melanogaster*. The "embryonic" sheet shows the hatching rates of eggs overexpressed by the actin driver (Supplementary Fig. 13). The "postembryonic" sheet shows the sex ratio of third-instar larvae and adults overexpressed by the actin driver (Supplementary Fig. 14).
